# A mammalian mirtron miR-1224 promotes tube-formation of human primary endothelial cells by targeting anti-angiogenic factor epsin2

**DOI:** 10.1038/s41598-017-05782-3

**Published:** 2017-07-17

**Authors:** Eiko Sakai, Yusuke Miura, Emi Suzuki-Kouyama, Kengo Oka, Masashi Tachibana, Kenji Kawabata, Fuminori Sakurai, Hiroyuki Mizuguchi

**Affiliations:** 1 Laboratory of Biochemistry and Molecular Biology, Graduate School of Pharmaceutical Sciences, Osaka University, 1-6 Yamadaoka, Suita, Osaka, 565-0871 Japan; 2 Laboratory of Hepatocyte Regulation, National Institute of Biomedical Innovation, Health and Nutrition, 7-6-8 Saito, Asagi, Ibaraki, Osaka, 567-0085 Japan; 3 Laboratory of Stem cell Regulation, National Institute of Biomedical Innovation, Health and Nutrition, 7-6-8 Saito Asagi, Ibaraki, Osaka, 565-0085 Japan; 4 iPS Cell-Based Research Project on Hepatic Toxicity and Metabolism, Graduate School of Pharmaceutical Sciences, Osaka University, 1-6 Yamadaoka, Suita, Osaka, 565-0871 Japan; 5 Global Center for Advanced Medical Engineering and Informatics, Osaka University, 2-2 Yamadaoka, Suita, Osaka, 565-0871 Japan

## Abstract

Angiogenesis, new vessel formation from pre-existing vessels, is a highly conserved event through vertebrates. However, the system for tuning angiogenesis by species-intrinsic factors is totally unknown. miR-1224 is a member of mammal-specific mirtrons, which were identified as non-canonical microRNAs. We found that the expression of miR-1224 was upregulated in capillary-like tube-forming human umbilical vein endothelial cells on Matrigel. Enforced expression of miR-1224 stimulated tube formation, whereas repression of endogenous miR-1224 inhibited formation. Enforced expression of miR-1224 enhanced VEGF signaling and repressed NOTCH signaling. The adaptor protein of clathrin-dependent endocytosis, epsin2, which has been shown to be a suppressor of angiogenesis, was a direct target of miR-1224. Knockdown of EPN2 stimulated tube formation, while overexpression of EPN2 repressed miR-1224-mediated stimulation. Our findings indicate that miR-1224 is a mammal specific modulator of angiogenesis.

## Introduction

Angiogenesis is the formation of new blood vessels from preexisting vessels. During angiogenesis, VEGF and Notch signaling play key roles in angiogenic sprouting. At angiogenic sprouts, ‘tip’ cells localized at the very front of sprouts segregate from the ‘stalk’ cells forming the stem of the vessel^1, 2^. Tip cells highly express VEGFR2 and Notch ligand Dll4. Vascular endothelial growth factor A (VEGF-A), a strong proangiogenic factor secreted from surrounding cells, binds to VEGFR2 on endothelial cells (ECs) of blood vessels, inducing dimerization and autophosphorylation of VEGFR2^3–5^ to initiate signaling cascades^6–8^. VEGF-A also induces the expression in ECs of Delta-like ligand 4 (DLL4), which suppresses sprouting by activating NOTCH on adjacent cells as its ligand^9, 10^, to form the stalk cells via reducing VEGFR2 expression. Thus, increased VEGF signaling enhances sprouting activity of ECs, while Notch signaling stabilizes ECs. These are highly conserved systems among species.

Signals of VEGF-A and NOTCH are controlled by clathrin-mediated endocytosis. Upon binding to VEGF-A, VEGFR2 undergoes clathrin-mediated endocytosis and is regulated by an endosomal trafficking-dependent process^11, 12^. DLL4 binding to the Notch-extracellular domain (NECD) initiates the sequential proteolytic cleavages of the Notch receptor, resulting in the release of the transcriptionally active Notch-intracellular domain (NICD) into the cytosol^13, 14^. Endocytosis of NECD into adjacent ligand-expressing cells is critical for NICD production^15^. Epsin1 and Epsin2 are involved in clathrin-mediated endocytosis^16–18^ and their defects caused deregulated activation of VEGF signaling. Mice with endothelial cell-specific deletion of both epsins have shown reduced tumor growth with overproduction of nonfunctional vessels^19^. Inhibitory peptides for the interaction between VEGFR2 and epsins stimulated angiogenesis both *in vitro* and *in vivo* ^20^.

MicroRNAs are a class of endogenous, noncoding small RNAs (18–24 nucleotides) that work for the post-transcriptional regulation of gene expression. Maturation of canonical miRNAs is mediated by two RNase III endonucleases: Dicer^21–23^ and Drosha^24–26^. After maturation, miRNAs bind by pairing to the 3′UTR of mRNAs, leading to either translational repression or degradation of the target mRNA^27–29^. The 5′ region of microRNAs (seed sequence) is important for the pairing. Mirtrons are derived from short hairpin introns and bypass Drosha cleavage by using the spliceosome to generate their precursors (pri-miRNAs)^30, 31^ and mirtrons have often evolved in species-specific ways^31–34^.

miR-1224 is a mammalian mirtron encompassed in the last intron of von Willebrand factor A domain containing 5B2 (VWA5B2) gene, and its function has been shown only in macrophages^35^. In this study, we found a proangiogenic function of miR-1224 in *in vitro* angiogenesis using human umbilical vein endothelial cells (HUVECs) and epsin2 as a direct target of mir-1224, providing a novel player for angiogenic modulation specific to particular mammals.

## Results

### miR-1224 expression is upregulated during *in vitro* tube formation

Angiogenesis is an evolutionarily conserved event driven by highly conserved angiogenic factors. miRNAs are relatively new biological regulators and their expression level could modulate the expression of basic angiogenic regulators. Therefore, we examined the miRNA expression profile during angiogenesis by microarray analysis to uncover a modulatory function of miRNAs for general angiogenesis. HUVECs form capillary-like structures called tubes when cultured on plates coated with Matrigel, an extracellular matrix secreted from mouse sarcoma cells. By comparing the miRNA signatures of P-HUVEC (HUVECs grown on regular culture plates) and M-HUVEC (HUVECs grown on Matrigel-coated plates), we found that 56 miRNAs were upregulated more than four-fold (Table S1). Several of them have been reported to be involved in EC functions (miR-139^36^ and miR-663^37, 38^) or in angiogenesis (miR-483^39^, miR-708^40^, miR-205^41^ and miR-296^42^). Interestingly, three mirtrons (miR-877, miR-1224, miR-1225) were included. These mirtrons are conserved in a mammalian-specific way^33^, raising the possibility that these mirtrons have modulating functions during mammalian angiogenesis. As none of these mirtrons has been reported to regulate any endothelial or angiogenic function, they are potentially novel proangiogenic miRNAs. Unfortunately, we found miR-1225 was hardly detected in northern blot analysis despite of high level of signal intensity in microarray analysis. Therefore, we started with miR-1224 in this study among the rest of two to elucidate the regulation of vessel formation by mammalian-specific mirtrons.

**Figure 1:**
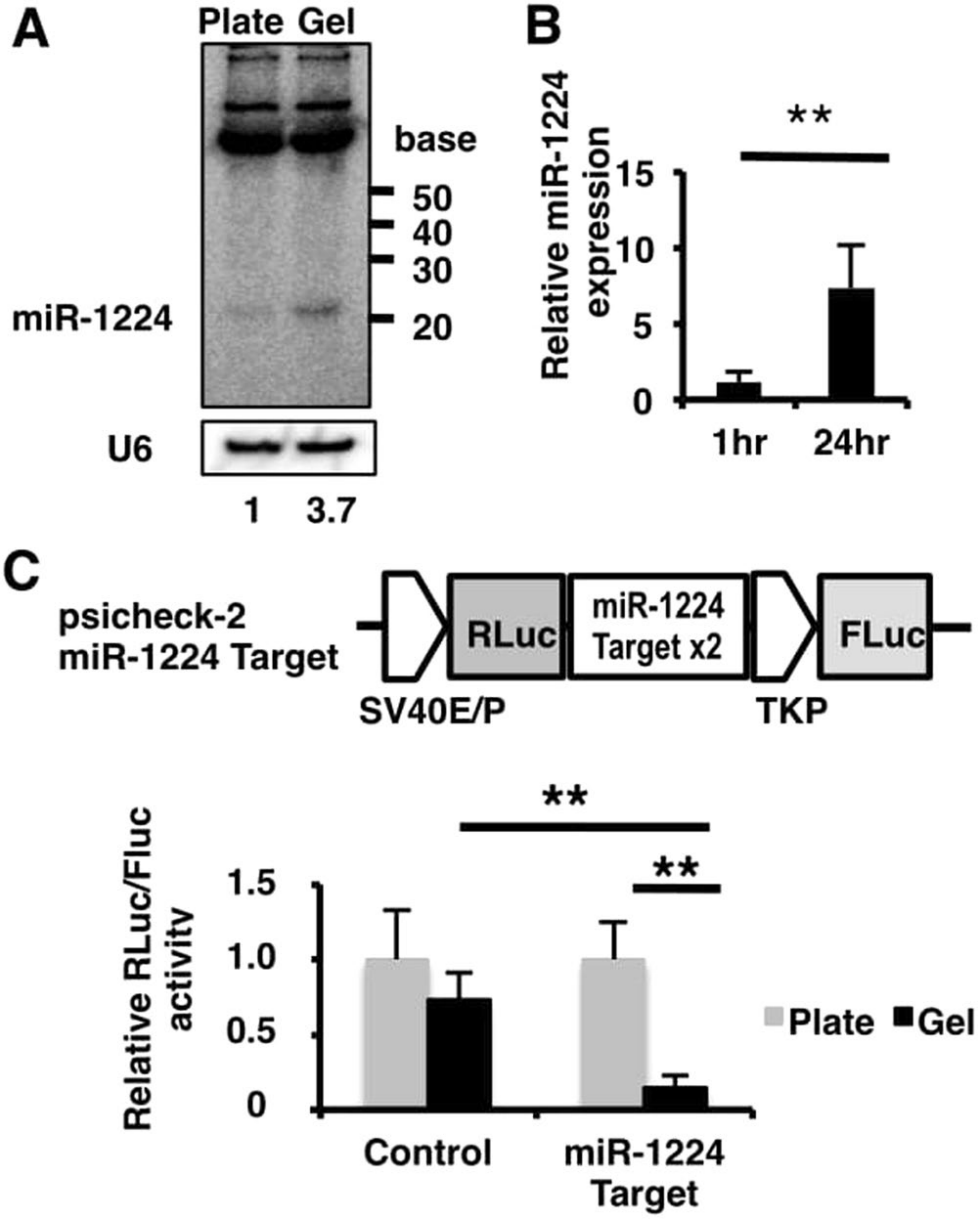
miR-1224 expression was upregulated during *in vitro* angiogenesis. (**A**) Northern blot analysis of miR-1224 expression in P-HUVECs (Plate) and M-HUVECs (Gel). The relative signal intensity of M-HUVECs to P-HUVECs is indicated. Uncropped versions of gels are provided in Supplementary Information. (**B**) qRT-PCR analysis of miR-1224 expression in M-HUVECs. Fold expression was calculated relative to one hour. Bars show the means ± S.D. with **p < 0.01 (n = 3). (**C**) Schematic diagram of the sensor plasmid (upper panel). Reporter assay in sensor plasmid–transfected HUVECs. Normalized renilla-luciferase levels (Rluc/Fluc) in M-HUVECs are shown as relative values to P-HUVECs (lower panel). Bars show the means ± S.D. with **p < 0.01 (n = 3).

First, we validated the increased expression of miR-1224 after tube formation. miR-1224 was detected at the expected size (~22nt) by northern blot analysis, and its signal intensity was 3.7-fold stronger in M-HUVEC than in P-HUVEC (Fig. 1A). The quantitative analysis of mature miR-1224 by quantitative reverse transcription polymerase chain reaction (qRT-PCR) further confirmed that miR-1224 expression was several-fold higher at 24 hours after culture onset on Matrigel compared to that at one hour after onset (Fig. 1B). The upregulation of miR-1224 was further confirmed by measuring post-transcriptional silencing activity using reporter constructs. The renilla-luciferase “sensor” reporter containing two complementary sites for miR-1224 was transfected into HUVECs. They were subsequently cultured on either culture plates or Matrigel-coated plates. This analysis revealed that the expression from the reporter containing miR-1224 target sites was repressed in M-HUVEC compared to the expression in P-HUVEC, indicating that functional miR-1224 was actually upregulated in M-HUVEC (Fig. 1C).

### miR-1224 stimulates *in vitro* angiogenesis

**Figure 2:**
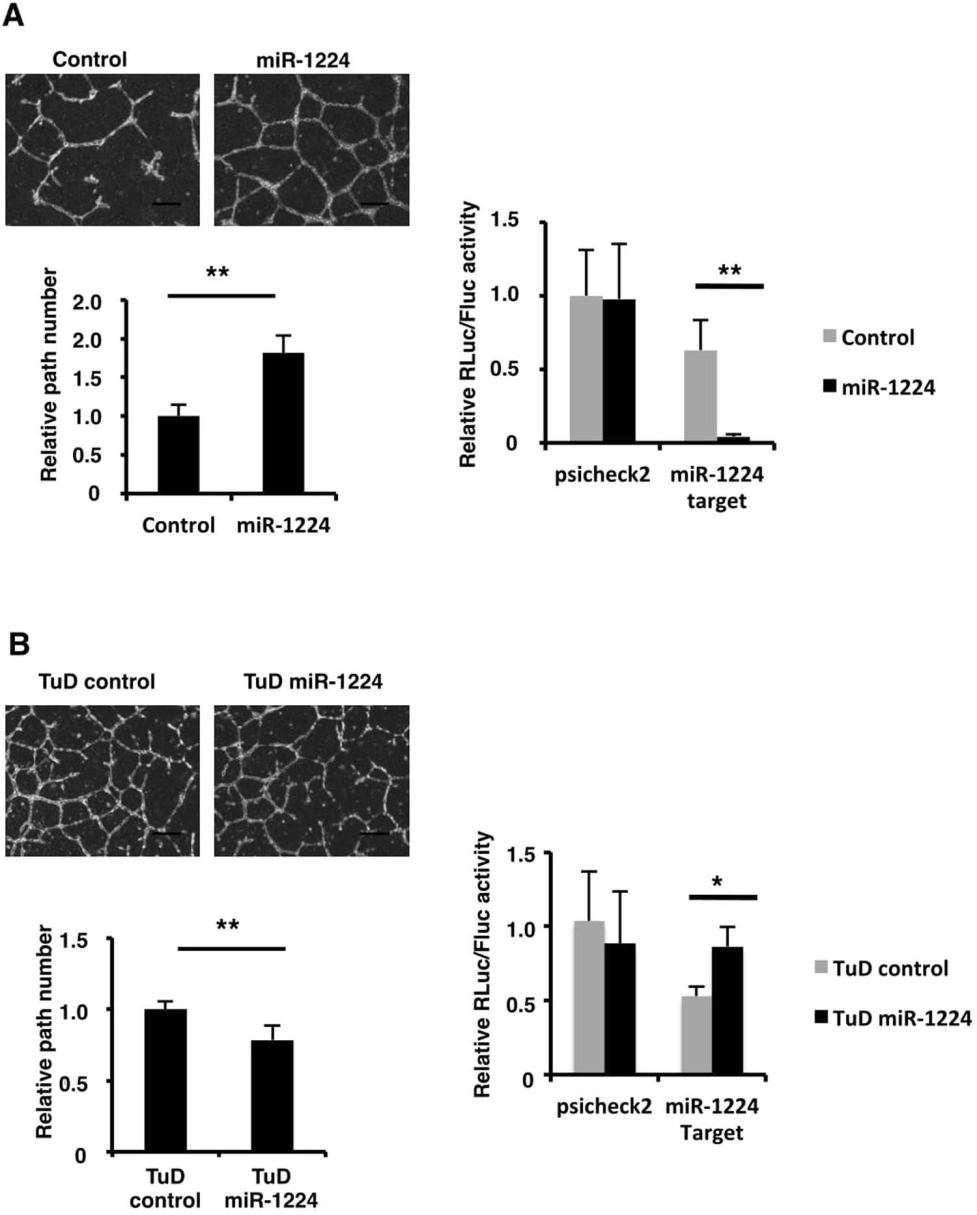
miR-1224 stimulated, and its inhibitor repressed, tube formation of HUVECs. (**A**) Tube formation assay of HUVECs transfected with miR-1224 mimics (left panels). Scale bars, 300 μm. Number of branches is shown. Bars show the means ± S.D. with **p < 0.01 (n = 4). Reporter assay in HUVECs co-transfected with miR-1224 mimics and sensor plasmids (right panel). Normalized renilla-luciferase levels (Rluc/Fluc) relative to the control plasmid with control mimics are shown. Bars show the means ± S.D. with **p < 0.01 (n = 3). (**B**) Tube formation assay of HUVECs transfected with miR-1224 TuD (left panels). Scale bars, 300 μm. Number of branches is shown. Bars show the means ± S.D. with **p < 0.01 (n = 4). Reporter assay in HUVECs co-transfected with TuD and sensor plasmids (right panel). Normalized renilla-luciferase levels (Rluc/Fluc) relative to the control plasmid with the control TuD are shown. Bars show the means ± S.D. with *p < 0.05 (n = 3).

Increased expression of miR-1224 in M-HUVEC suggested that miR-1224 positively regulated *in vitro* tube formation. To verify this, HUVECs were transfected with a miR-1224 mimic and then cultured on Matrigel. Capillary-like network formation was clearly enhanced in the miR-1224-transfected cells, compared to the control mimic-transfected cells. The number of branches of microvessels in the miR-1224-transfected cells increased by approximately two-fold compared to that in the control mimic-transfected cells (Fig. 2A). Conversely, tube formation was inhibited following the transfection of miR-1224-Tough Decoy (TuD)^43^, which is a double-stranded RNA oligonucleotide designed to bind to and inhibit endogenous miR-1224 molecules (Fig. 2B). The activity of the transfected exogenous miR mimic or TuD was examined using the sensor plasmids. The decrease in the luciferase activity in the control mimic or TuD-transfected cells indicates endogenous miR-1224 activity. Much further decrease in the miR-1224 mimic-transfected cells was observed compared to the control mimic and significant increase was observed in the TuD-miR-1224-transfected cells, confirming that both miR-1224 mimic and TuD-miR-1224 functionally control the miR-1224 activity in the cells (Fig. 2). Taken together, these results indicate that miR-1224 stimulates tube formation *in vitro*.

### miR-1224 modulates NOTCH/VEGF signaling

**Figure 3:**
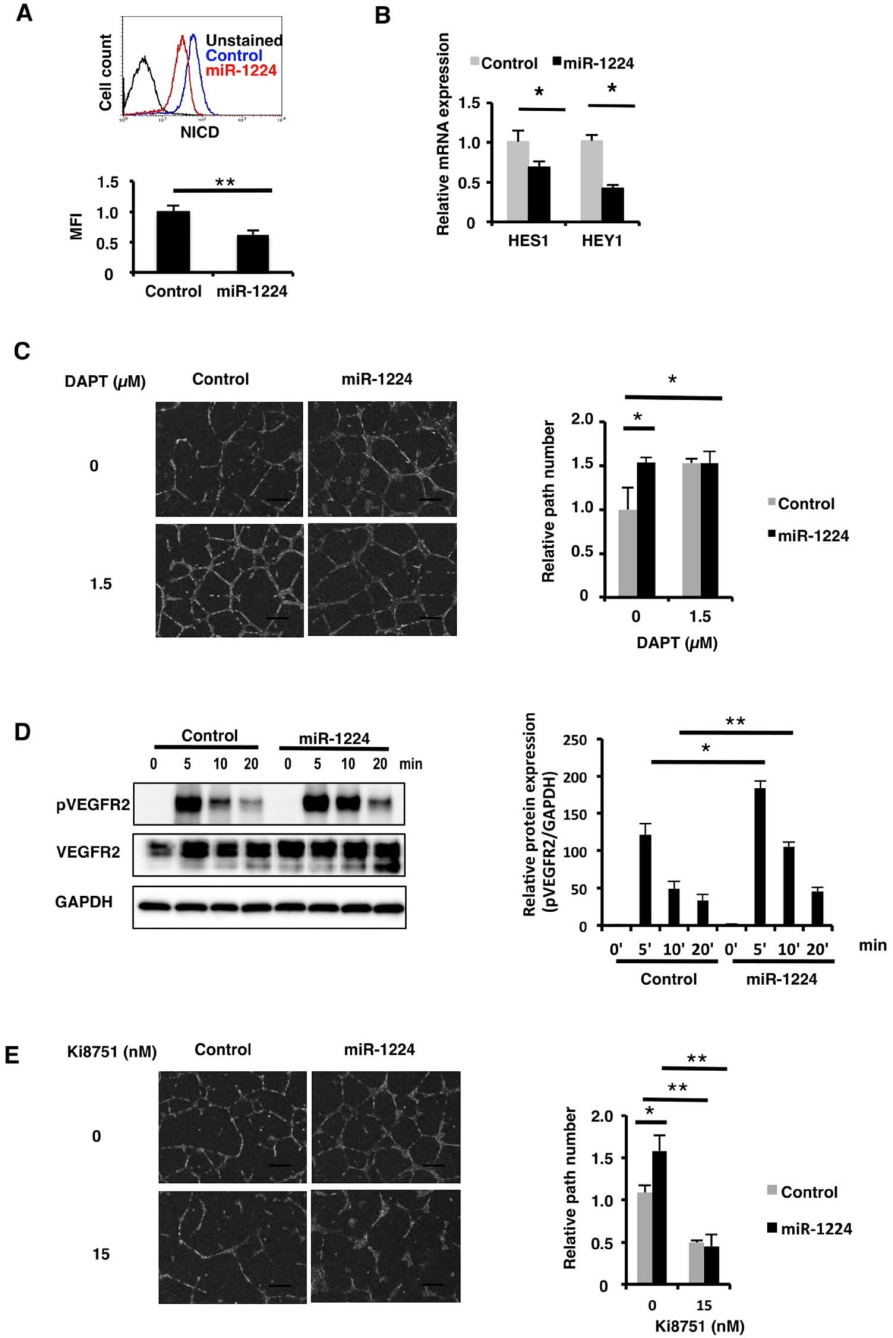
Reduced NOTCH signaling and elevated VEGF signaling in miR-1224–transfected HUVECs. (**A**) FACS analysis of HUVECs transfected with miR-1224 (red) or control mimic (blue) for the cleaved NICD. Unstained control (black) is shown. Mean fluorescence intensity (MFI) was calculated. Bars show the means ± S.D. with **p < 0.01 (n = 4). (**B**) qRT-PCR analysis of HES1 and HEY1 mRNA. Relative expression to negative control mimic is shown. Bars show the means ± S.D. with *p < 0.05 (n = 3). (**C**) Tube formation assay of HUVECs transfected with miRNA mimics in the presence of NOTCH inhibitor (DAPT). Scale bars, 300 μm. Relative number of branches is shown compared to the control mimic-transfection without inhibitor. Bars show the means ± S.D. with **p < 0.01 (n = 3). (**D**) Western blot analysis of phosphorylated VEGFR2 in HUVECs stimulated with VEGF-A. Uncropped versions of gels are provided in Supplementary Information. Results from three independent experiments were analyzed and shown. Bars show the means ± S.E. with *p < 0.05, **p < 0.01 (n = 3). (**E**) Tube formation assay of HUVECs transfected with miRNA mimics in the presence of VEGFR2 inhibitor (Ki18751). Scale bars, 300 μm. Relative number of branches is shown compared to the control mimic-transfection without inhibitor. Bars show the means ± S.D. with *p < 0.05, **p < 0.01 (n = 3).

Next, to explore the mechanism by which miR-1224 stimulates tube formation, we examined the influence of increased miR-1224 expression on major angiogenic signaling through VEGF and NOTCH. In cultured human ECs, activation of NOTCH signaling has been shown to inhibit new sprout formation and migration^9, 10^. The activation of NOTCH signaling leads to a proteolytic cleavage to release NICD into the cytoplasm. NICD translocates to the nucleus and mediates the transcriptional regulation of a variety of genes involved in vascular development. To explore whether miR-1224 affects EC behavior by modulating NOTCH, the level of NICD was assessed by intracellular flow cytometry using an antibody specifically recognizing NICD. It revealed a significant decrease of NICD in miR-1224-transfected cells compared to control cells (Fig. 3A). Consistently, expression of NOTCH target genes, HES1 and HEY1^44, 45^, was downregulated in miR-1224-transfected HUVECs (Fig. 3B). These results indicated that NOTCH signaling was negatively regulated by miR-1224.

To evaluate the effects of the decrease in NOTCH activity on the stimulatory activity of miR-1224 to tube formation, HUVECs were cultured on Matrigel in the presence of DAPT, a ɣ-secretase inhibitor. DAPT stimulated tube formation in the control mimic-transfected cells. In the miR-1224-transfected cells, tube formation was stimulated in the absence of DAPT and no further stimulation was observed in the presence of DAPT suggesting that the stimulatory function of miR-1224 was replaced by NOTCH inhibition to a similar extent (Fig. 3C).

VEGFR2 is the main signal transducer of VEGF for angiogenesis. Therefore, it was also possible that activated VEGFR2 was elevated by miR-1224 expression in HUVECs. To investigate this, miR-1224-transfected HUVECs were starved for 12 hours followed by treatment with VEGF, and phosphorylated VEGFR2 was analyzed by immunoblotting (Fig. 3D). VEGFR2 phosphorylation was significantly enhanced at 5 and 10 minutes after stimulation in miR-1224-transfectants. To examine the functional relevance of the VEGFR2 activation by miR-1224, the tube formation assay was performed in the presence of VEGFR2 specific inhibitor Ki8751. The stimulation by miR-1224 was suppressed in the presence of Ki8751. These results indicate that miR-1224 stimulated the tube formation through VEGFR2 activation (Fig. 3E).

In total, miR-1224 suppressed the NOTCH signal pathway and activated VEGF signaling to regulate tube formation.

### EPN2 mRNA is a direct target of miR-1224

In order to determine the target genes responsible for miR-1224-induced upregulation of the tube formation, we predicted the target genes of miR-1224 *in silico* using two sequence-based target prediction software programs, miRanda and TargetScan. Each program yielded a large number of genes as possible miR-1224 targets. The 162 candidate genes commonly predicted by both methods were listed (Table S2). Among them, EPN2 was particularly interesting to us for two reasons. First, the double-inactivation of endothelial *Epn1* and *Epn2* resulted in elevated and prolonged VEGFR2 phosphorylation as well as in the stabilization of VEGFR2 protein, leading to heightened VEGF/VEGFR2 signaling and excessive tumor angiogenesis in mice ^17, 46^. Second, it has been shown that inactivation of *Epn1* and *Epn2* resulted in decreased NOTCH signaling in *in vivo* knockout experiments^47^. Our observations in Fig. 3 are highly reminiscent of these previous reports, suggesting that miR-1224 might target the EPN2 mRNA and downregulate its expression.

**Figure 4:**
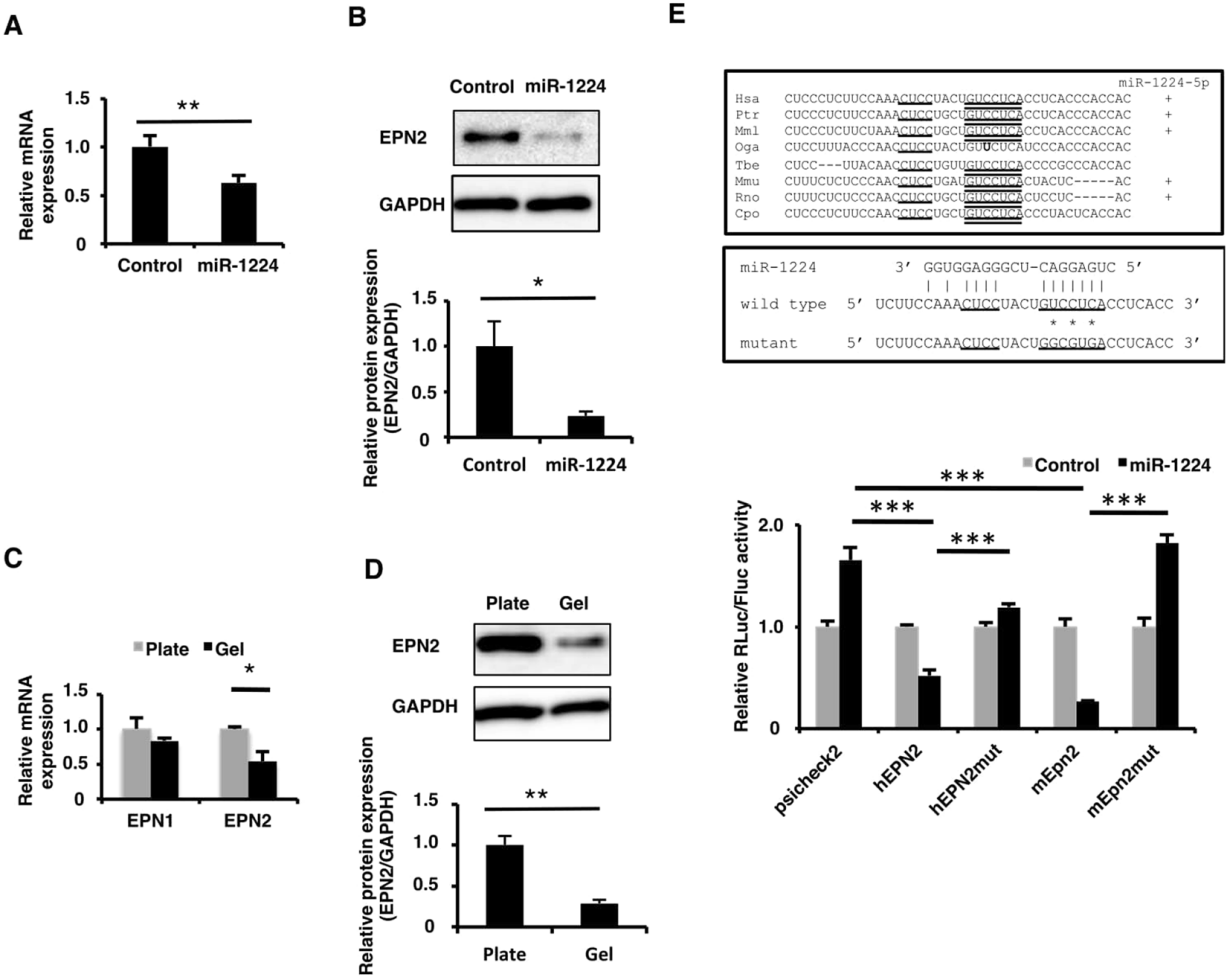
EPN2 expression was suppressed by miR-1224 through its 3′-UTR. (**A**) qRT-PCR analysis of EPN2 mRNA in HUVECs transfected with miR-1224 mimic. Relative expression to the negative control mimic is shown. Bars show the means ± S.D. with **p < 0.01 (n = 3). (**B**) Western blot analysis of EPN2 protein in HUVECs transfected with miR-1224 mimic. Uncropped versions of gels are provided in Supplementary Information. The normalized signal intensities were shown as relative values to the control. Bars show the means ± S.E. with *p < 0.05 (n = 3). (**C**) qRT-PCR analysis of EPN1 and EPN2 mRNA in M-HUVECs. Relative expression to P-HUVECs is shown. Bars show the means ± S.D. with *p < 0.05 (n = 3). (**D**) Western blot analysis of EPN2 protein in M- and P-HUVECs. Uncropped versions of gels are provided in Supplementary Information. Relative expression to P-HUVECs is shown. Bars show the means ± S.E. with **p < 0.01 (n = 3). (**E**) Sequence alignment of 3′-UTRs containing the miR-1224 seed-matched sequence. Seed and additional matched sequences are underlined. Species that have miRBase-registered miR-1224-5p are indicated by + in the right column (top panel). Sequence alignment of miR-1224 and the target region of 3′-UTRs. Asterisks indicate the mutated nucleotides (middle panel). Reporter assay was performed in HEK293 cells (bottm panel). Normalized renilla-luciferase activity (Rluc/Fluc) relative to the control empty psiCHECK2 is shown. Bars show the means ± S.D. with ***p < 0.001 (n = 3).

To explore whether miR-1224 regulates the endogenous EPN2, HUVECs were transfected with a control mimic or miR-1224 mimic, and endogenous EPN2 expression was assessed. As expected, the introduction of miR-1224 mimic to HUVECs resulted in significant (~50%) downregulation of EPN2 mRNA as well as EPN2 protein (Fig. 4A and B).

Next, we asked whether endogenous EPN2 expression was suppressed in M-HUVEC where miR-1224 was upregulated. Both mRNA and protein levels of EPN2 were downregulated in M-HUVEC compared to P-HUVEC (Fig. 4C and D). These observations were consistent with the notion that elevated expression of miR-1224, as shown in Fig. 1, caused the suppression of EPN2 expression in M-HUVEC.

The target sequence of miR-1224 was identified in many mammalian *EPN2* genes (Fig. 4E). To determine whether miR-1224 directly regulates EPN2 expression, fragments from the 3′-UTR of human *EPN2* and mouse *Epn2* were cloned downstream from a *renilla-luciferase* gene in reporter plasmids. miR-1224 overexpression repressed the luciferase activity both of the reporter plasmid containing the 3′-UTR fragment of human *EPN2* or mouse *Epn2* gene but not the control empty reporter. Furthermore, point mutations of the seed-matched sequence prevented repression by miR-1224. These results indicated that both human and mouse EPN2 mRNAs are direct targets of miR-1224 through binding to the seed-matched sequences in the 3′-UTRs. Taken together, these observations suggested that miR-1224 negatively regulates EPN2 expression by directly targeting its 3′-UTR.

### Knockdown of EPN2 enhances *in vitro* angiogenesis

**Figure 5:**
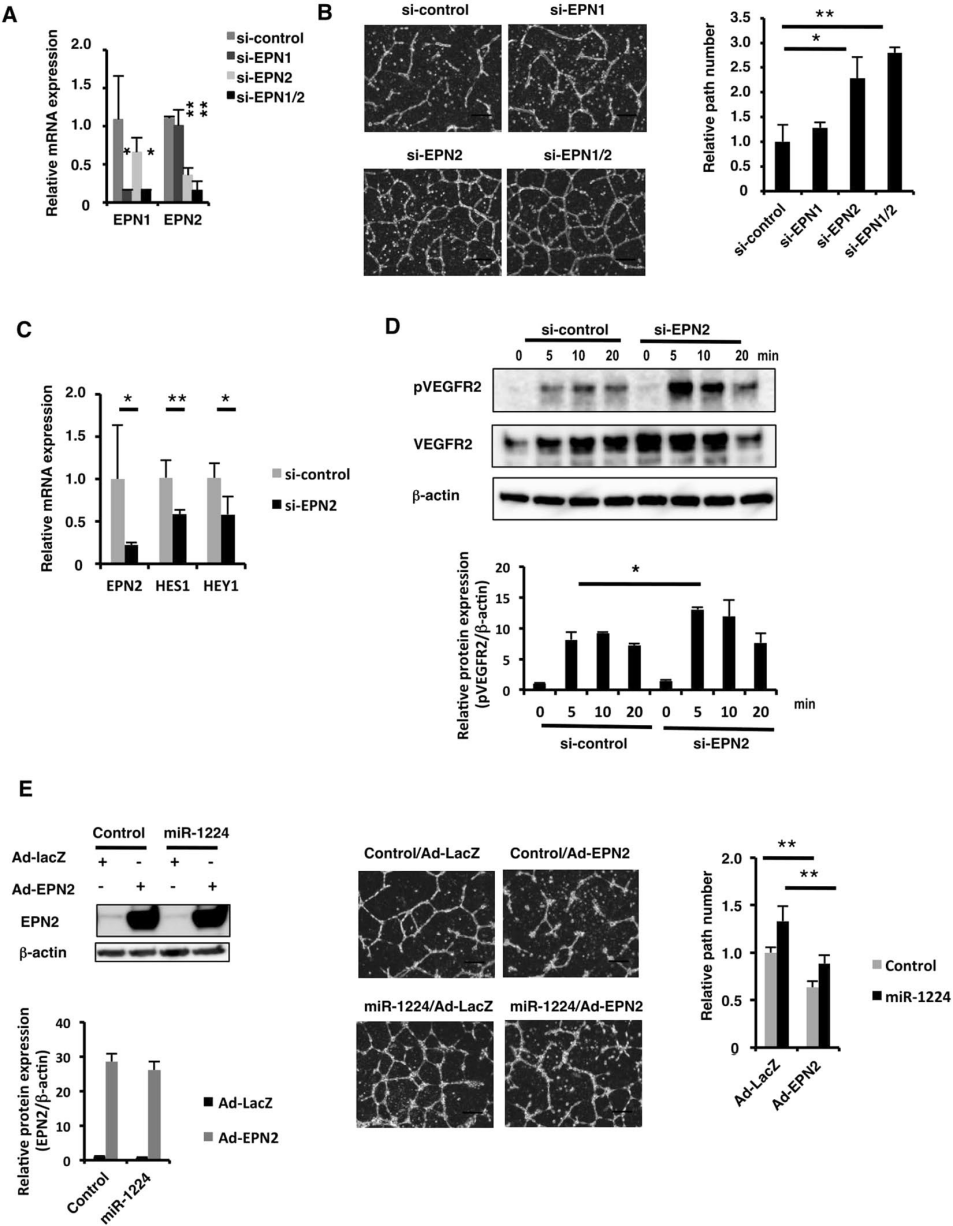
Knockdown of EPN2 stimulated tube formation. (**A**) qRT-PCR analysis of EPN1 and EPN2 mRNA in siRNA-transfected HUVECs. Relative expression to the control siRNA is shown. Bars show the means ± S.D. with *p < 0.05, **p < 0.01 (n = 3). (**B**) Tube formation assay of siRNA-transfected HUVECs. Scale bars, 300 μm. Relative path number to the control siRNA-transfection is shown. Bars show the means ± S.D. with *p < 0.05, **p < 0.01 (n = 3). (**C**) qRT-PCR analysis of HES1 and HEY1 mRNA in siRNA-transfected HUVECs. Relative expression to the control siRNA is shown. Bars show the means ± S.D. with *p < 0.05, **p < 0.01 (n = 3). (**D**) Western blot analysis of phosphorylated VEGFR2 in HUVECs stimulated with VEGF-A. Uncropped versions of gels are provided in Supplementary Information. The normalized signal intensities were shown as relative values to the control siRNA. Bars show the means ± S.E. with *p < 0.05 (n = 3). (**E**) Tube formation assay of HUVECs transfected with miR-1224 and transduced with EPN2-expressing Ad vector. EPN2 protein expression was analyzed by western blotting (left). Uncropped versions of gels are provided in Supplementary Information. The normalized signal intensities were shown as relative values to the control mimic with the control LacZ-expressing Ad vector. Bars show the means ± S.E. (n = 3).

To determine whether the reduced expression of EPN2, likely caused by elevated expression of miR-1224, could contribute to the stimulation of tube formation in our experiments, EPN2 and EPN1 expression was knocked down by small interfering RNA (siRNA) independently or together in HUVECs (Fig. 5A). Since mouse Epn1 and Epn2 were reported to have complementary roles^47^, a single knockdown of human EPN2 was assumed to have no effects. However, capillary networking was unexpectedly stimulated by a single knockdown of EPN2 (Fig. 5B), indicating EPN2 by itself had a significant suppressive function. Combined knockdown of EPN1 and EPN2 stimulated tube formation more than either EPN1 or EPN2 single knockdown, which is consistent with *in vivo* knockout experiments^47^ (Fig. 5A and B).

In order to examine that suppression of EPN2 has effects on NOTCH and VEGFR signals as in miR-1224-transfectd cells, expression of NOTCH target genes and the phosphorylation of VEGFR2 were examined. mRNA expression of HES 1 and HEY1 was significantly downregulated and the phosphorylation of VEGFR2 was stimulated in siEPN2-transfected HUVECs (Fig. 5C and D). These results indicated that NOTCH and VEGF signals were positively and negatively regulated by EPN2, respectively. This is consistent to the notion that the miR-1224’s effects on these signals were mediated by suppressing its target gene *EPN2*. To verify this notion, an overexpression experiment was performed next.

HUVECs were transfected with miR-1224 mimics and subsequently transduced with EPN2- or control LacZ-expressing adenovirus (Ad) vectors. Exogenous EPN2 expression level was twenty-fold more than the endogenous EPN2 (Fig. 5E). Tube formation of the control mimic-transfected cells was suppressed by transduction with the EPN2-expressing Ad vector as expected. Importantly, the EPN2-expressing Ad vector suppressed tube formation in miR-1224-transfected cells to a similar extent to control, indicating that the function of miR-1224 was mediated by suppression of EPN2 expression at least in part (Fig. 5E).

Taken together, our studies demonstrated that: 1) miR-1224 induces angiogenesis by upregulating VEGF signaling and downregulating NOTCH signaling; 2) miR-1224 promotes angiogenesis by targeting EPN2, a negative regulator of angiogenesis; 3) therefore a novel modulation of angiogenesis by a species-specific mechanism, miR-1224/EPN2 pathway, was identified.

## Discussion

Angiogenesis is a fundamental event that is required during development. Clathrin-mediated endocytosis, which regulates angiogenesis through the regulation of VEGF^11, 12^ and NOTCH signals^15^, is also fundamental to the regulation of the internalization and trafficking of membrane-anchored molecules. Epsins are an evolutionarily conserved protein family that facilitate clathrin-mediated internalization of ubiquitinated cell surface receptors^18, 48–50^. Since epsins are widely used in various processes through yeast^51^ to vertebrates and worms^47, 52, 53^, and since their cargo-specificity has not been identified so far, epsins could be considered fundamental proteins that simply mediate signals from outer environments to intracellular machineries. Alternatively, specific regulation of endocytic components could affect sensitivity to environmental stimuli by regulating endocytosis of receptors and ligands at appropriate timing.

Mirtrons have gained attention as an interesting tool for exploring unidentified regulatory networks. However, because they are expressed at much lower levels than canonical miRNAs, it might be expected that mirtrons have only modest roles compared to canonical miRNAs^54^. However, numerous sequencing analyses have clearly shown that a number of mirtrons have been strictly conserved through evolution in a species-specific manner, as among Drosophila, among mammals and even among primates^30–34^, indicating that they have evolved for some biologically relevant functions related to species-specific characteristics. On the other hand, their lower expression in turn suggests that mirtrons might be induced under fleeting conditions but not in a steady state, so that they could function with pinpoint accuracy rather than in homeostatic regulation. In this study, the P-HUVEC showed lower expression of miR-1224 compared with M-HUVEC, and the increase in miR-1224 in either endogenous or exogenous mode was associated with a higher capacity for tube formation in these cells. Our study revealed the proangiogenic function of miR-1224 in an *in vitro* system where miR-1224 expression was induced during angiogenesis. This finding clearly indicated that temporally regulated expression of miR-1224 modulates basic angiogenesis. Furthermore, this finding suggested that miR-1224 could be a specific regulator of angiogenesis depending on the expression of both miR-1224 and targetable EPN2. Therefore, it is interesting that the targetable sequence in the 3′UTR of EPN2 gene seems to appear only in the species that belong to Euarchontogliras in the Supplementary Figure S1. How and why the miR-1224/EPN2 pathway was acquired in these species remains elusive.

Studies on mammal-specific regulation of angiogenesis also could be valuable for the blood vessel pathology. For example, angiogenesis plays a critical role for the onset of gestational hypertension that can lead to a serious condition called preeclampsia. In this case, excess secretion of soluble VEGFR1 and soluble Endoglin from placenta suppresses the placental angiogenesis resulting in abnormal blood vessel formation and defects in blood circulation. It is conceivable that a mammal-specific angiogenic regulation system might co-evolve with the development of placenta and be involved in its pathology. Our finding that the miR-1224/EPN2 pathway was active during angiogenesis of umbilical veins is interesting from this point of view.

In summary, our approach to identify miRNAs involved in the regulation of angiogenesis revealed that a species-specific mirtron suppressed a highly conserved endocytic adaptor molecule, EPN2, resulting in the modulation of the basic angiogenic system. It suggested that the miR-1224/EPN2 system might be beneficial to the specification of some characteristics of those species. Further studies in this line of research would be required to fully understand how species-specific regulation systems have been established, presumably in part by species-specific mirtrons.

## Materials and Methods

### Cell cultures

HUVECs were purchased from Lonza (Walkersville, MD) and cultured in EGM-2 medium (Lonza). HEK293 cells (a transformed human embryonic kidney cell line) were cultured with Dulbecco’s modified Eagle’s medium (DMEM) supplemented with 10% fetal bovine serum (FBS), streptomycin (100 μg/ml), and penicillin (100 U/ml).

### Plasmids

The fragment containing the putative target sites for miR-1224 in the 3′-UTR of human EPN2 and mouse Epn2 was amplified using PCR with genomic DNA prepared from HepG2 cells (a human hepatocellular carcinoma cell line) and C57/B6Ncr mouse tail, respectively, as templates. The primers are shown in Table S3. The PCR products were inserted downstream from the *renilla-luciferase* gene in the psiCHECK-2 luciferase plasmid (Promega, Madison, WI). The sensor plasmid was constructed by insertion of double-stranded oligonucleotides perfectly matched to the miR-1224 sequence into psiCHECK-2. For the mutations (hEPN2mut and mEpn2mut), the seed sequences of the predicted miR-1224 target sites were mutated from GTCCTCA to GGCGTGA by site-directed mutagenesis^55^ with the oligonucleotides listed in Table S3. All constructs were verified by DNA sequencing. Adenovirus (Ad) vectors were constructed by an improved *in vitro* ligation method^56^. The EPN2-expressing plasmid, pHMCA-EPN2, was generated by inserting an EPN2 cDNA fragment, which had been cloned from cDNAs of HepG2 cells by PCR, into pHMCA5^57^. The chicken β-actin promoter with cytomegalovirus enhancer (CA) was a kind gift from Dr. J. Miyazaki (Osaka University, Osaka Japan). pHMCA-EPN2 was digested with I*-Ceu*I/PI*-Sce*I and ligated into I*-Ceu*I/PI*-Sce*I–digested pAdHM15-RGD^57^, resulting in pAdHM15-RGD-CA-EPN2. Ad-EPN2 was generated by transfection of PacI-linearized pAdHM15-RGD-CA-EPN2 into HEK293 cells and purified as described previously^57^. The CA-driven β-galactosidase (LacZ)-expressing Ad vector, AdLacZ, was generated previously^57^. The biological virus titer was determined using Adeno-X Rapid Titer Kit (Clontech).

### Tough Decoy (TuD)

Tough Decoy was designed according to a previous study^43^. Negative control 2′*–O-*methylated RNA and anti-miR-1224 2′*–O-*methylated RNA sense and antisense oligonucleotides were synthesized by Fasmac (Kanagawa, Japan). The RNA sequences are shown in Table S3.

### Transfection and adenovirus vector transduction

HUVECs were transfected with miRIDIAN microRNA Mimics-miR-1224, miRIDIAN microRNA Mimic Negative Control #1 (Thermo Fisher Scientific, Lafayette, CO), siGENOME SMART pool (Thermo Fisher Scientific) (10 nM), and Tough Decoy (50 nM) with Lipofectamine RNAiMAX reagent (Invitrogen Life Technologies, Carlsbad, CA). For co-transfection experimants, HUVECs were transfected with reporter plasmids (50 ng) and microRNA mimics (10 nM) or TuDs (50 nM) using Lipofectamine 2000 reagent (Invitrogen Life Technologies) and analyzed 48 hours after transfection. For Ad vector transduction, Ad vectors (multiplicity of infection (MOI) of 40) were added to culture media and washed out after four hours’ incubation and analyzed for RNA and protein levels and subjected to tube formation assay 48 hours after transduction.

### Matrigel tube formation assay

HUVECs (passages < 12, 1.5 × 10^5^/0.5 ml) were seeded in a 24-well plate and transfected with either microRNA mimics (10 nM), siRNAs (10 nM) or Tough Decoy (50 nM) on the next day. Forty-eight hours after transfection, tube formation assay was performed. HUVECs were plated (6.5 × 10^4^ cells/0.5 ml) into Matrigel-coated wells in a 24-well plate to induce tube networking. BD matrigel matrix was purchased from BD Biosciences (Bedford, MA). Twenty-four hours later, the wells were examined with an Olympus CKX41 microscope and images were captured using an Olympus DP20–5 digital camera. The capillary tubes in those images were counted. For analysis of TuD transfection, images were taken eight hours after transfection instead of 24 hours after. For inhibitor assays, Ki8751 (Calbiochem, San Diego, CA) or N–[(3,5-difluorophenyl) acetyl]-L-alanyl-2-phenyl-1,1-dimethylethyl ester-glycine (DAPT; Peptide Institute) was added during tube formation on Matrigel at the indicated concentrations. To recover cells from Matrigel-coated wells, the cells were washed with PBS and incubated with 1 ml of ice-cold BD Cell Recovery Solution (BD Biosciences) with agitation on ice until the gel was resolved. Collected cells were washed with PBS twice and subjected to further analysis.

### VEGF stimulation

HUVECs transfected with either miRNA mimics or siRNA (10 nM) were cultured for 48 hours, starved for 12 hours with DMEM, and stimulated with VEGF-A (50 ng/ml). VEGF signaling was analyzed by examination of the phosphorylated VEGFR2 by western blotting. Signal intensity of phosphorylated VEGFR2 was normalized by GAPDH. Normalized value of the control at 0 min was set to 1.

### MicroRNA microarray analysis

Total RNA samples (100 ng) were extracted from P-HUVECs and M-HUVECs. Hybridization and signal acquisition of Agilent human miRNA microarray (Release 10.1) (Agilent Technologies, Santa Clara, CA) carrying 723 human and 76 human viral miRNAs was performed using an Agilent DNA Microarray Scanner with Agilent ScanControl version 7.0 software. The analysis and background correction were performed using Agilent Feature Extraction Software ver. 9.5. All the microarray data in this work is deposited at GEO with an accession number of GSE56663

### Flow cytometry analysis

HUVECs (1 × 10^6^) were fixed in 1% paraformaldehyde (PFA)/PBS followed by permeabilization with 0.1% Triton X-100. After washing with staining buffer (PBS/1%FBS), the cells were incubated with mouse anti-NOTCH intracellular domain monoclonal antibody conjugated with phycoerythrin (PE) (mN1A, eBioscience, San Diego, CA) at 4 °C for 30 min and washed twice with staining buffer. The intracellular NICD was quantified by immunofluorescence on a FACSCalibur flow cytometer (BD Biosciences).

### Real-Time RT-PCR

Total RNA was isolated from the transfected cells 48 hours after transfection with the use of ISOGEN (Nippon Gene, Tokyo, Japan). cDNA was synthesized using SuperScript II reverse transcriptase (Invitrogen Life Technologies) and a random primer. Quantitative polymerase chain reaction (qPCR) was performed using StepOnePlus real-time PCR system with FAST SYBR Green Master Mix (Applied Biosystems, FosterCity, CA). The sequences of the primers used in this study are listed in the Table S3. The values were normalized by GAPDH expression. miR-1224 in the isolated RNA was determined by quantitative reverse transcription PCR (qRT-PCR). cDNAs were synthesized using miRScript II RT Kit (Qiagen) and were subjected to qPCR using the miRScript SYBR Green PCR Kit (Qiagen) and primers specific to U6 and miR-1224. The values were normalized by U6. The primer sequences were as follows: miR-1224-5p, 5′-gtgaggactcgggaggtgg-3′; U6, 5′-gcaaattcgtgaagcgttcc-3′.

### Northern blot analysis

The small RNA fraction was isolated from total RNA from P-HUVECs (Plate) and M-HUVECs (Gel) 24 hours after plating using the miRVana miRNA Isolation Kit (Ambion, Foster City, CA), and 5 μg of RNAs was subjected to 12.5% PAGE/2 M urea to resolve and was subsequently subjected to northern blotting. Blots were hybridized with a ^32^P-labelled locked nucleic acid (LNA)-oligonucleotide probe to detect miR-1224 as previously described^58^. The LNA probe was synthesized by Gene Design (Osaka, Japan). The signal intensity of mature miR-1224 was normalized with that of U6.

### Western blot analysis

Cells were lysed in RIPA buffer (20 mM Tris pH 7.4, 300 mM NaCl, 1% Triton X-100, 0.1% SDS, 1% sodium deoxycholate) containing proteinase inhibitors (Sigma Aldrich, St. Louis, MO) and phosphatase inhibitors (Nacalai Tesque, Kyoto, Japan). Whole cell lysates (20 μg of protein) were subjected to SDS–PAGE and western blotting. Blots were incubated with the following antibodies: mouse anti-KDR monoclonal (1:100) (23B31, IBL, Gunma, Japan), rabbit anti-phospho-VEGFR2 (pY1175) (1:1000) (19A10, Cell Signaling Technology, Danvers, MA), rabbit anti-Epsin2 (1:1000) (PA5-12090, Fisher Thermo Scientific), mouse anti-β-actin (1:5000) (AC-15, Sigma Aldrich) and rabbit anti-G3pdh (1:2000) (2275-PC-1, Trevigen, Gaithersburg, MD).

### Target prediction and Dual luciferase reporter assay

Two microRNA target site prediction methods, TargetScan Human (Release 5.1) and miRanda^59^, were used to predict hsa-miR-1224-5p target candidates and putative binding sites in the 3′-untranslated region (3′-UTR) of each gene. HEK293 cells were transfected with 30 ng of dual luciferase reporter plasmids and 10 nM of miRIDIAN microRNA Mimics in 24-well plates with Lipofectamine 2000. Forty-eight hours after transfection, dual luciferase assays were performed using the Dual luciferase reporter assay system (Promega) according to the manufacturer’s instructions. Renilla-luciferase activity was first normalized using firefly-luciferase expression control. For each reporter construct, the co-transfectant with a negative control mimic was 1.

### Statistical analysis

Statistical significance was determined using Student’s *t*-test. Data are presented as the means ± S.E. for western blot analysis and means ± S.D. for others.

## Electronic supplementary material

Supplementary information
