# Supplementary material for: A mammalian mirtron miR-1224 promotes tube-formation of human primary endothelial cells by targeting anti-angiogenic factor epsin2
Source: Sci Rep. 2017 Jul 17;7:5541. doi: 10.1038/s41598-017-05782-3 (PMC5514154; doi:10.1038/s41598-017-05782-3)
Supplement: Supplementary file 1 — Supplementary information [file 41598_2017_5782_MOESM1_ESM.pdf]

A mammalian mirtron miR-1224 promotes tube-formation of human primary endothelial cells by targeting anti-angiogenic factor epsin2.

Eiko Sakai[1], Yusuke Miura[1], Emi Suzuki-Kouyama[3], Kengo Oka[1], Masashi Tachibana[1], Kenji Kawabata[3], Fuminori Sakurai[1] and Hiroyuki Mizuguchi[1,2,4,5*]

Table S1 List of microRNA upregulated in M-HUVEC.			
miRNA	Plate	Matrigel	Ratio
hsa-miR-1225-5p	435	54171	124.53
hsa-miR-371-5p	51	4643	91.04
hsa-miR-877	9	619	68.78
hsa-miR-188-5p	61	4223	69.23
hsa-miR-139-3p	17	531	31.24
hsa-miR-769-3p	10	272	27.20
hsa-miR-936	6	168	28.00
hsa-miR-623	11	287	26.09
hsa-miR-557	10	141	14.10
hsa-miR-601	7	169	24.14
hsa-miR-630	43	989	23.00
hsa-miR-30c-1*	4	78	19.50
hsa-miR-887	34	571	16.79
hsa-miR-134	50	846	16.92
hsa-miR-650	4	65	16.25
hsa-miR-671-5p	45	693	15.40
hsa-miR-483-5p	43	627	14.58
hsa-miR-422a	7	107	15.29
hsa-miR-1228*	2	28	14.00
hsa-miR-663	45	593	13.18
hsa-miR-135a*	48	588	12.25
hsa-miR-760	9	102	11.33
hsa-miR-125a-3p	41	457	11.15
hsa-miR-187*	9	91	10.11
hsa-miR-202	50	520	10.40
hsa-miR-149*	8	71	8.88
hsa-miR-708	5	49	9.80
hsa-miR-486-5p	16	148	9.25
hsa-miR-886-5p	2	20	10.00
hsa-miR-518c*	5	45	9.00
hsa-miR-373*	10	83	8.30
hsa-miR-498	16	129	8.06
hsa-miR-638	448	3540	7.90

hsa-miR-212	21	160	7.62
hsa-miR-345	12	97	8.08
hsa-miR-610	5	39	7.80
hsa-miR-665	6	41	6.83
hsa-miR-629*	17	126	7.41
hsa-miR-542-5p	11	79	7.18
hsa-miR-602	11	68	6.18
hsa-miR-509-3-5p	8	52	6.50
hsa-miR-1226*	28	166	5.93
hsa-miR-296-5p	9	50	5.56
hsa-miR-575	121	678	5.60
hsa-miR-490-5p	6	36	6.00
hsa-miR-339-3p	8	44	5.50
hsa-miR-622	9	48	5.33
hsa-miR-572	57	298	5.23
hsa-miR-205	11	55	5.00
hsa-miR-125b-2*	13	65	5.00
hsa-miR-564	13	61	4.69
hsa-miR-1224-5p	78	376	4.82
hsa-miR-193b*	10	44	4.40
hsa-miR-582-3p	2	10	5.00
hsa-miR-138-2*	5	24	4.80

* mirtrons are highlighted by grey colour

Table S2. Predicted target gene set.	
Gene	Description
ABHD13	abhydrolase domain containing 13
ADCK4	aarF domain containing kinase 4
ADCY2	adenylate cyclase 2 (brain)
AHCYL1	adenosylhomocysteinase-like 1
ALDH1A3	aldehyde dehydrogenase 1 family, member A3
ALPK3	alpha-kinase 3
AMBRA1	autophagy/beclin-1 regulator 1
AMMECR1L	AMME chromosomal region gene 1-like
AQP8	aquaporin 8
ARC	activity-regulated cytoskeleton-associated protein
ARF6	ADP-ribosylation factor 6
ARHGEF11	Rho guanine nucleotide exchange factor (GEF) 11
ARHGEF9	Cdc42 guanine nucleotide exchange factor (GEF) 9
ARID4B	AT rich interactive domain 4B (RBP1-like)
ASF1A	ASF1 anti-silencing function 1 homolog A (S. cerevisiae)
ATRX	alpha thalassemia/mental retardation syndrome X-linked
BAZ2A	domain adjacent to zinc finger domain, 2A
BCL6	B-cell CLL/lymphoma 6
CASC3	cancer susceptibility candidate 3
CBLN2	cerebellin 2 precursor
CCDC97	coiled-coil domain containing 97
CD200R1	CD200 receptor 1
CDK6	cyclin-dependent kinase 6
CLDND1	claudin domain containing 1
CNTN4	contactin 4
COL18A1	collagen, type XVIII, alpha 1
COL5A2	gen, type V, alpha 2
COPB1	coatomer protein complex, subunit beta 1
COPS7A	COP9 constitutive photomorphogenic homolog subunit 7A (Arabidopsis)
CPLX2	complexin 2
CPNE1	copine I

CRLF3	cytokine receptor-like factor 3
CSNK1G1	casein kinase 1, gamma 1
DCP2	DCP2 decapping enzyme homolog (S. cerevisiae)
DCUN1D4	DCN1, defective in cullin neddylation 1, domain containing 4 (S. cerevisiae)
DDR1	discoidin domain receptor tyrosine kinase 1
DDX3X	DEAD (Asp-Glu-Ala-Asp) box polypeptide 3, X-linked
DES	desmin
DLX1	distal-less homeobox 1
DNAJC14	DnaJ (Hsp40) homolog, subfamily C, member 14
DRP2	dystrophin related protein 2
DYRK1A	dual-specificity tyrosine-(Y)-phosphorylation regulated kinase 1A
E2F8	E2F transcription factor 8
ELAVL2	ELAV (embryonic lethal, abnormal vision, Drosophila)-like 2 (Hu antigen B)
EPN2	epsin 2
EYA4	eyes absent homolog 4 (Drosophila)
FGF13	fibroblast growth factor 13
FIGN	fidgetin
FNDC3B	fibronectin type III domain containing 3B
FNIP1	liculin interacting protein 1
FRMD4A	FERM domain containing 4A
GALC	galactosylceramidase
GLCCI1	glucocorticoid induced transcript 1
GLUD1	glutamate dehydrogenase 1
GTDC1	glycosyltransferase-like domain containing 1
GTF2E1	general transcription factor IIE, polypeptide 1, alpha 56kDa
GTF2H1	general transcription factor IIH, polypeptide 1, 62kDa
H3F3B	H3 histone, family 3B (H3.3B)
HCN3	hyperpolarization activated cyclic nucleotide-gated potassium channel 3
HMGN3	high mobility group nucleosomal binding domain 3
HNRNPH2	heterogeneous nuclear ribonucleoprotein H2 (H')
HOXC13	homeobox C13

HOXD9	homeobox D9
HRH1	histamine receptor H1
HTRA3	HtrA serine peptidase 3
HYAL1	hyaluronoglucosaminidase 1
IGF2R	insulin-like growth factor 2 receptor
INHBE	inhibin, beta E
INSM2	insulinoma-associated 2
INSR	insulin receptor
IPO11	importin 11
IPO5	importin 5
ITGAV	integrin, alpha V (vitronectin receptor, alpha polypeptide, antigen CD51)
KANK2	KN motif and ankyrin repeat domains 2
KAT2B	K(lysine) acetyltransferase 2B
KCNA1	potassium voltage-gated channel, shaker-related subfamily, member 1 (episodic ataxia with myokymia)
KCNK3	potassium channel, subfamily K, member 3
KCTD1	potassium channel tetramerisation domain containing 1
KLF3	Kruppel-like factor 3 (basic)
KPNA1	karyopherin alpha 1 (importin alpha 5)
LANCL1	LanC lantibiotic synthetase component C-like 1 (bacterial)
LARP7	La ribonucleoprotein domain family, member 7
LMNB1	lamin B1
LRRC41	leucine rich repeat containing 41
MED20	mediator complex subunit 20
MEIS2	Meis homeobox 2
METAP1	methionyl aminopeptidase 1
MGLL	monoglyceride lipase
MKRN1	makorin ring finger protein 1
MNT	MAX binding protein
MPP2	membrane protein, palmitoylated 2 (MAGUK p55 subfamily member 2)
MYBPHL	myosin binding protein H-like
NFIB	lear factor I/B
NICN1	nicolin 1

NKAIN2	Na+/K+ transporting ATPase interacting 2
NTN1	netrin 1
NUFIP2	nuclear fragile X mental retardation protein interacting protein 2
ODF4	outer dense fiber of sperm tails 4
OLFM1	olfactomedin 1
OSBP	oxysterol binding protein-like 10
PANX1	pannexin 1
PDK2	pyruvate dehydrogenase kinase, isozyme 2
PEX5	peroxisomal biogenesis factor 5
PIGR	polymeric immunoglobulin receptor
PKD1	polycystic kidney disease 1 (autosomal dominant)
PLCD4	phospholipase C, delta 4
PLN	phospholamban
PPP1R9B	protein phosphatase 1, regulatory (inhibitor) subunit 9B
PPP2R5A	protein phosphatase 2, regulatory subunit B', alpha
PRKACB	protein kinase, cAMP-dependent, catalytic, beta
PRKAG3	protein kinase, AMP-activated, gamma 3 non-catalytic subunit
ProSAPiP1	ProSAPiP1 protein
PRPF4	PRP4 pre-mRNA processing factor 4 homolog (yeast)
PRPF4B	PRP4 pre-mRNA processing factor 4 homolog B (yeast)
PSME4	proteasome (prosome, macropain) activator subunit 4
PTP4A2	protein tyrosine phosphatase type IVA, member 2
PTPRF	protein tyrosine phosphatase, receptor type, F
RAP2A	RAP2A, member of RAS oncogene family
RAP2C	RAP2C, member of RAS oncogene family
REPS2	RALBP1 associated Eps domain containing 2
RHOQ	ras homolog gene family, member Q
RND3	Rho family GTPase 3
RNF146	ring finger protein 146
RPP25	ribonuclease P/MRP 25kDa subunit
RPS6KA5	ribosomal protein S6 kinase, 90kDa, polypeptide 5
RUNX1T1	runt-related transcription factor 1; translocated to, 1 (cyclin D-related)
S1PR1	sphingosine-1-phosphate receptor 1

SCN3B	sodium channel, voltage-gated, type III, beta
SDCCAG8	serologically defined colon cancer antigen 8
SEMA4G	sema domain, immunoglobulin domain (Ig), transmembrane domain (TM) and short cytoplasmic domain, (semaphorin) 4G
SEMA5A	transmembrane domain (TM) and short cytoplasmic domain, (semaphorin)
SESTD1	SEC14 and spectrin domains 1
SLC16A2	solute carrier family 16, member 2 (monocarboxylic acid transporter 8)
SLC16A7	te carrier family 16, member 7 (monocarboxylic acid transporter 2)
SLC25A22	solute carrier family 25 (mitochondrial carrier: glutamate), member 22
SLC29A2	solute carrier family 29 (nucleoside transporters), member 2
SLC9A5	solute carrier family 9 (sodium/hydrogen exchanger), member 5
SMCR7L	Smith-Magenis syndrome chromosome region, candidate 7-like
SMG5	smg-5 homolog, nonsense mediated mRNA decay factor (C. elegans)
SNPH	syntaphilin
SNX19	sorting nexin 19
SOBP	sine oculis binding protein homolog (Drosophila)
SOX9	SRY (sex determining region Y)-box 9
SP1	Sp1 transcription factor
ST8SIA3	8 alpha-N-acetyl-neuraminide alpha-2,8-sialyltransferase 3
SYNGR2	synaptogyrin 2
TAC4	tachykinin 4 (hemokinin)
TARBP2	TAR (HIV-1) RNA binding protein 2
TMEM104	transmembrane protein 104
TMEM30B	transmembrane protein 30B
TNKS	tankyrase, TRF1-interacting ankyrin-related ADP-ribose polymerase

TOP1	topoisomerase (DNA) I
TPP1	tripeptidyl peptidase I
UBE2V1	ubiquitin-conjugating enzyme E2 variant 1
UBE3B	ubiquitin protein ligase E3B
UBR7	ubiquitin protein ligase E3 component n-recognin 7 (putative)
UNC119B	unc-119 homolog B (C. elegans)
VEZF1	vascular endothelial zinc finger 1
XPO1	exportin 1 (CRM1 homolog, yeast)
ZBTB44	nc finger and BTB domain containing 44
ZDHHC6	zinc finger, DHHC-type containing 6
ZHX1	zinc fingers and homeoboxes 1

Table S3　Oligonucleotide sequences

Name	Sequence
cDNA cloning	
EPN2 forward	5'CGACCTCGAGGCCGCCatgacgacttcgtctatcag acggc 5'
EPN2 reverse	5'GGTGGCGGCCGCctagagaaggaaagggttggttg 3'
	* Linker sequences for restriction sites are in capital letters.
Primer sets for qRT-PCR	
EPN1 forward	5' cggagatcaaggttcgagag 3'
EPN1 reverse	5' tactccatcagcgtcatggc 3'
EPN2 forward	5' gcctccactaaccagaccaac 3'
EPN2 reverse	5' gattgtacggtggctccagtg 3'
HPRT1 forward	5' ctgaggatttggaaagggtg 3'
HPRT1 reverse	5' aatccagcaggtcagcaaag 3'
HES1 forward	5' gacattctggaaatgacagtgaag 3'
HES1 reverse	5' attgatctgggtcatgcagttg 3'
HEY1 forward	5' attgagaagcgccgacgagacc 3'
HEY1 reverse	5' tttccctcctgccgtatgcagc 3'
GAPDH forward	5' ggtggtctcctctgacttcaaca 3'
GAPDH reverse	5' gtggtcgttgagggcaatg 3'
Tough Decoy	
miR-1224 TuD top strand	5'gacggcgcuaggaucaucaacccaccucccaaguccucacc aaguauucuggu 3'
miR-1224 TuD bottom strand	5'accagaauacaacccaccucccaaguccucaccaagaugau ccuagcgccguc 3'
Negative control TuD top strand	5'gacggcgcuaggaucaucaacuaucgcgaguaucgacguc gaggcccaaguauucuggu 3'
Negative control TuD bottom strand	5'accagaauacaacuaucgcgaguaucgacgucgaggccca agaugauccuagcgccguc 3'
Reporter plasmids	
mouse Epn2 forward	5' caactaatcccttttttgtacctgg 3'

mouse Epn2 reverse	5' ggtctcttaactcaagatttaatac 3'
human EPN2 forward	5' tccaaactcctactgtcctcacctc 3'
human EPN2 reverse	5' gctgtcatgttggggcctcaaagtc 3'
human EPN2 mutation forward	5' tccaaactcctactgtcctcacctc 3'
mouse Epn2 mutation forward	5' ctgatggcgtgactactcactgttaagaaaagt 3'
mouse Epn2 mutation reverse	5' gagtagtcacgccatcaggaggttgggagagaa 3'
miR-1224 complemantary sense strand	5'tcgagCTCCACCTCCCCAGTCCTCAcagcCTCCACCTCCCCAGTCCTCAcgttt 3'
miR-1224 complemantary antisense strand	5'aaacGTGAGGACTGGGGAGGTGGAGgctgGTGAGGACTGGGGAGGTGGAGc 3'<br>* Sequences corresponding for miR-1224-5p are in capital letters.
Probe for Norther blot analysis	
antisense-LNA miR-1224-5p	5' c<u>a</u>cc<u>t</u>ccc<u>g</u>a<u>gt</u>cc<u>t</u>c<u>a</u> 3'
antisense U6	5' tgctaaatcttctctgtatcgt 3'<br>* The LNA-modified nucleotides are underlined.

```
                               mir-1224-5p seed-matched sequence
Hsa   CUCCCUCUUCCAA---------ACUCCUACUGUCCUCACCU----------------CACCCACCAC
Ptr   CUCCCUCUUCCAA---------ACUCCUGCUGUCCUCACCU----------------CACCCACCAC
Mml   CUCCCUCUUCUAA---------ACUCCUGCUGUCCUCACCU----------------CACCCACCAC
Oga   CUCCUUUACCCAA---------CCUCCUACUGUUCUCAUCC----------------CACCCACCAC
Tbe   CUCC---UUACAA---------CCUCCUGUUGUCCUCACCC----------------CGCCCACCAC   Euarchontaglires
Mmu   CUUUCUCUCCCAA---------CCUCCUGAUGUCCUCACUA----------------CUC-----AC
Rno   CUUUCUCUCCCAA---------CCUCCUGCUGUCCUCACUC----------------CUC-----AC
Cpo   CUCCCUCUUCCAA---------CCUCCUGCUGUCCUCACCC----------------UACUCACCAC
Ocu   CACCCUCUCCUGA---------GGUCCUACUGUCCCCCCCC----------------CCC-CA-CAC
Sar   CC--UCUUCCACC---------CCUCCUGCUUUUCCUCCCC----------------AACCCUCCAC
Eeu   CU--CCUUCCUCCAAUGCCCAGCCUCCUGCUCCCUCCCCCA----------------AGCC-----C
Cfa   CU--CCUUCCCAA---------UUUCCUGCUUUCCUUGCCC----------------CACUCACCAC   Laurasiatheria
Fca   --CCCCCCCCCA---------CCUCCUGCUUCCCCC-CGC----------------CCCCCACCAC
Eca   CACCCUCUCCCCA-------------------------CCC----------------GACCCACCAC
Bta   CU------CCCUC---------UCCCCUGCUUCCUUUGUCC----------------CACCCACCAC
Dno   -------------------------------------------------------------------   Xenartha
Laf   CUCCCUCUCCCAG---------CCUCCUCCUUUCCUCGCCC----------------CACCAGCCUC
Ete   CUCCGUCUUACAG---------CUUCUUGCCUUCCUCAUCCAUGCCCCUACCCACAUUCCCAGUCAA   Afrotheria
Mdo   -------------------------------------------------------------------
Oan   -------------------------------------------------------------------   non-placental
Aca   -------------------------------------------------------------------   non-mammal
Gga   -------------------------------------------------------------------
```

Supplementary figure S1 Sequence alignment of 3'UTR containing the mir-1224 target sequences. Seed-matched sequences are underlined.

Related to Fig. 1A

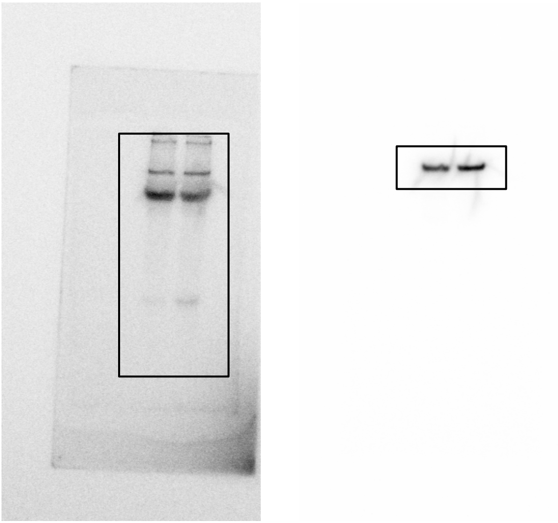

miR-1224 U6

Related to Fig. 3D

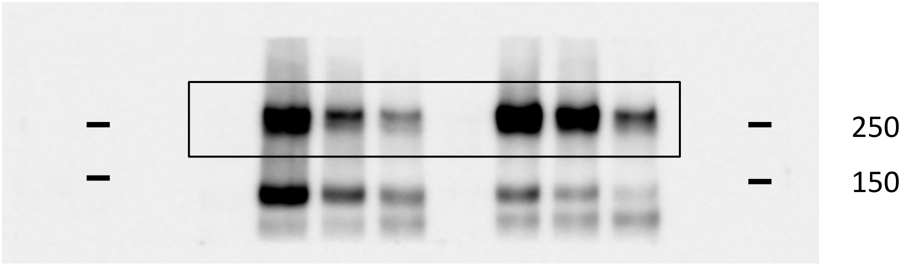

Anti-pVEGFR2

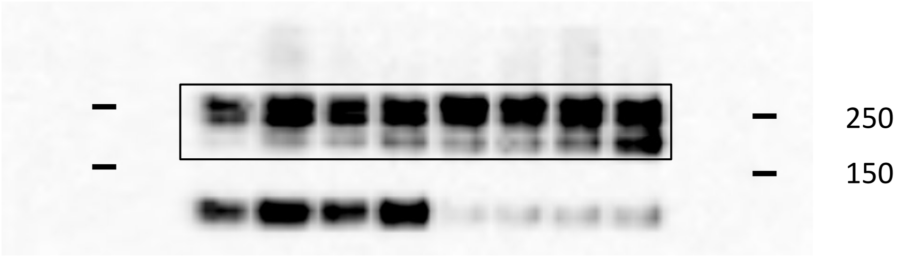

Anti-VEGFR2

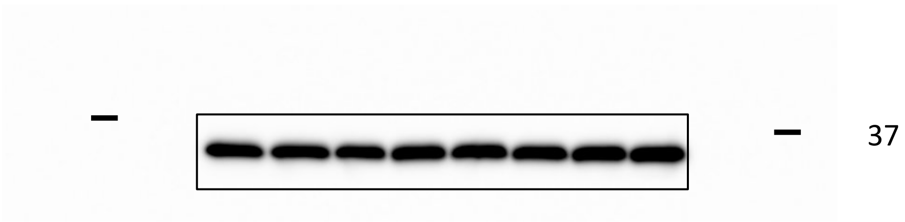

Anti-GAPDH

Related to Fig. 4B

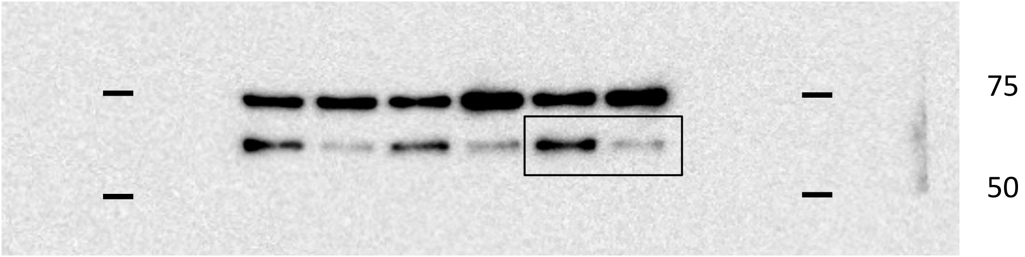

Anti-EPN2

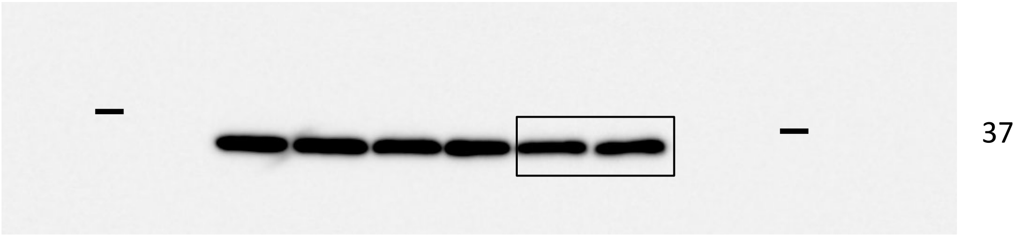

Anti-GAPDH

Related to Fig. 4D

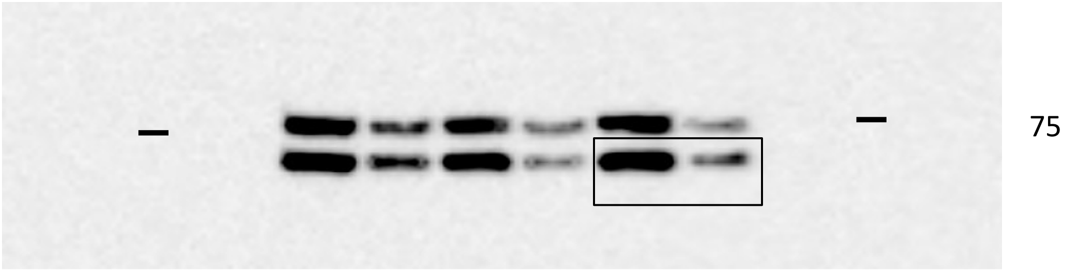

Anti-EPN2

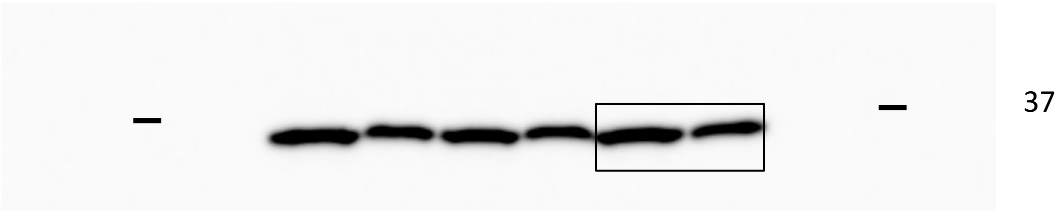

Anti-GAPDH

Related to Fig. 5D

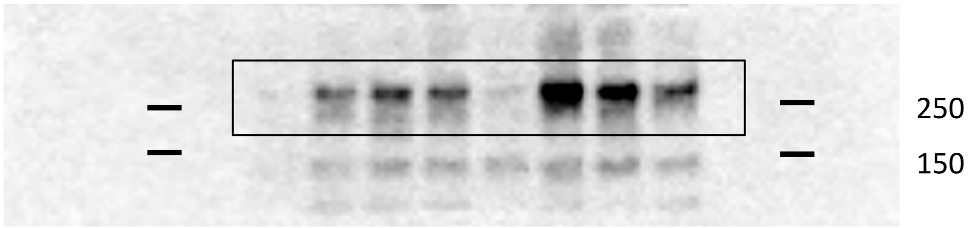

Anti-pVEGFR2

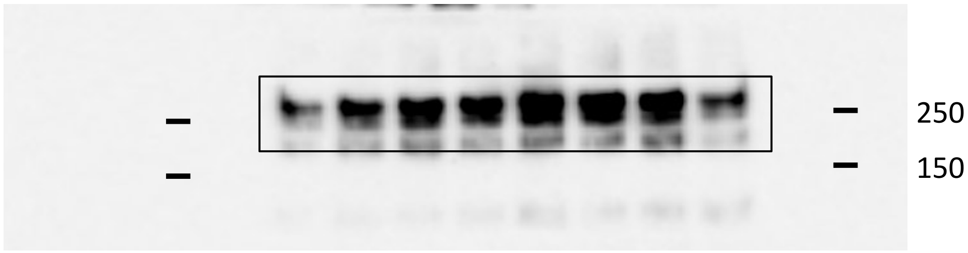

Anti-VEGFR2

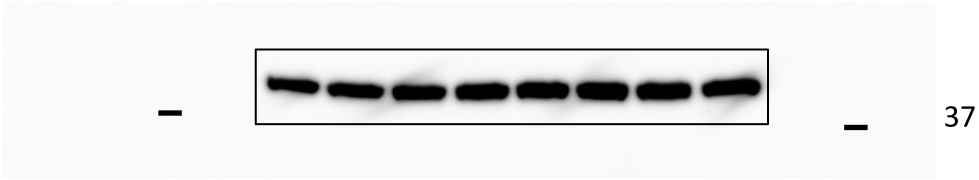

Anti-β-actin

Related to Fig. 5E

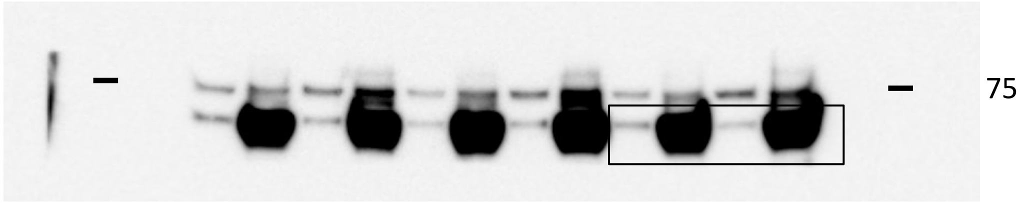

Anti-EPN2

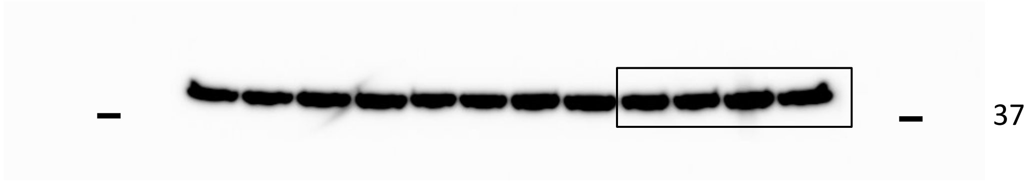

Anti-β-actin
